# Glucagon-like peptide-1 facilitates cerebellar parallel fiber glutamate release through PKA signaling in mice in vitro

**DOI:** 10.1038/s41598-023-34070-6

**Published:** 2023-05-16

**Authors:** Xin-Yuan Wang, Yang Liu, Li-Xin Cao, Yu-Zi Li, Peng Wan, De-Lai Qiu

**Affiliations:** 1 Department of Neurology, Affiliated Hospital of Yanbian University, Yanji, 133000 Jilin China; 2 Department of Physiology, College of Basic Medicine, Jilin Medical University, Jilin, 132013 Jilin China; 3 Department of Physiology and Pathophysiology, College of Medicine, Yanbian University, Yanji, 133000 Jilin China; 4 Department of Cardiology, Affiliated Hospital of Yanbian University, Yanji, 133000 Jilin China

**Keywords:** Neuroscience, Physiology

## Abstract

Glucagon-like peptide-1 (GLP-1) is mainly secreted by preproglucagon neurons; it plays important roles in modulating neuronal activity and synaptic transmission through its receptors. In the present study, we investigated the effects of GLP-1 on parallel fiber–Purkinje cell (PF-PC) synaptic transmission in mouse cerebellar slices using whole-cell patch-clamp recording and pharmacology methods. In the presence of a γ-aminobutyric acid type A receptor antagonist, bath application of GLP-1 (100 nM) enhanced PF-PC synaptic transmission, with an increased amplitude of evoked excitatory postsynaptic synaptic currents (EPSCs) and a decreased paired-pulse ratio. The GLP-1-induced enhancement of evoked EPSCs was abolished by a selective GLP-1 receptor antagonist, exendin 9–39, as well as by the extracellular application of a specific protein kinase A (PKA) inhibitor, KT5720. In contrast, inhibiting postsynaptic PKA with a protein kinase inhibitor peptide-containing internal solution failed to block the GLP-1-induced enhancement of evoked EPSCs. In the presence of a mixture of gabazine (20 μM) and tetrodotoxin (1 μM), application GLP-1 significantly increased frequency, but not amplitude of miniature EPSCs via PKA signaling pathway. The GLP-1-induced increase in miniature EPSC frequency was blocked by both exendin 9–39 and KT5720. Together, our results indicate that GLP-1 receptor activation enhances glutamate release at PF-PC synapses via the PKA signaling pathway, resulting in enhanced PF-PC synaptic transmission in mice in vitro. These findings suggest that, in living animals, GLP-1 has a critical role in the modulation of cerebellar function by regulating excitatory synaptic transmission at PF-PC synapses.

## Introduction

Glucagon-like peptide-1 (GLP-1) is mainly secreted by preproglucagon (PPG) neurons in the nucleus tractus solitarius of the brain^1^. The axons of PPG neurons project widely to regions such as the hypothalamic nucleus, amygdala, brainstem, and cerebellum. GLP-1 therefore plays critical roles in regulating neuronal activity and synaptic transmission through its receptors in the central nervous system^2,3^. GLP-1 receptors are G-protein coupled and are expressed in various brain regions, including the hypothalamus nuclei, hippocampus, amygdala, ventral tegmental area, substantia nigra, nucleus tractus solitarius, and cerebellum^4–10^.

GLP-1 regulates neuronal activity by modulating multiple ion channels such as calcium channels, non-selective cation channels, voltage-dependent potassium channels, and short transient receptor potential channel 5 (TRPC5)^11^. In rat nodose ganglion neurons, GLP-1 induces depolarization of the neuronal membrane potential and increases cytosolic calcium, thus resulting in an increased spike firing rate^12^. Furthermore, GLP-1 induces the inhibition of potassium channels, leading to depolarization and an increased firing rate in nodose neurons^13^. However, extracellular GLP-1 application evokes inward currents, which are accompanied by increased membrane conductance and the depolarization of corticotropin-releasing hormone neurons of the hypothalamic paraventricular nucleus (PVN)^8^. GLP-1 receptors are expressed in the bed nucleus of the stria terminalis; activation of these receptors induces bidirectional membrane potential changes in neurons of this brain region^10^. Moreover, GLP-1 receptor activation reduces the conductance of voltage-dependent potassium channels, resulting in the increased spontaneous discharge frequency of olfactory mitral cells^14^. It has also been reported that the optogenetic activation of PPG neurons produces a mix of excitation and inhibition that creates a multiphasic response, shaping the firing pattern of the olfactory bulb mitral cells; this finding suggests that PPG neurons may drive the neuromodulation of olfactory output and change the synaptic map that regulates olfactory coding^15^. The GLP-1-induced excitation of skullcap cells may also lead to changes in the excitability of the olfactory cortex and hypothalamus region by inhibiting the conductance of voltage-dependent potassium channels and enhancing the release of glutamate^16^. Furthermore, a GLP-1 receptor agonist can induce depolarization and an increased firing rate in arcuate nucleus neurons; this effect is prevented by the intracellular administration of a G-protein blocker^17^.

GLP-1 can regulate neuronal activity by modulating multiple signal transduction pathways, such as the mitogen-activated protein kinases (MAPKs) ERK1/2, protein kinase A (PKA), protein kinase C (PKC), and cytosolic calcium signal pathways^11,18^. The activation of GLP-1 receptors activates adenylate cyclase, resulting in increased intracellular cyclic adenosine monophosphate (cAMP); this further modulates neuronal signal transduction and gene transcription^19^. The application of GLP-1 receptor allosteric modulators can augment cAMP signaling and increase intracellular calcium concentration^20^, as well as induce AMP-activated protein kinase (AMPK) inhibition and MAPK activation of neurons through PKA signaling pathways^21^. In addition, GLP-1 receptor activation can enhance the frequency of miniature excitatory postsynaptic currents (mEPSCs) and decrease the paired-pulse ratio (PPR) via presynaptic α-amino-3-hydroxy-5-methyl-4-isoxazolepropionic acid (AMPA)/kainate receptors in medium spiny neurons, thus suggesting that GLP-1 receptors modulate glutamate synthesis and release^22^. The application of GLP-1 agonists transiently enhances γ-aminobutyric acid (GABA) type A receptor-mediated synaptic and tonic currents in CA3 pyramidal neurons of rat brain slices^23,24^. In contrast, GLP-1 receptor blockade increases spontaneous excitatory synaptic activities, differentially modulates voltage-gated and chemically gated calcium influx, and reduces pyramidal neuron overexcitation in hippocampal slices of 3 × transgenic Alzheimer’s disease model mice^25^. In the arcuate nucleus, a GLP-1 analog increases mEPSC frequency and evoked EPSC amplitude in half of neurons, but also increases miniature inhibitory postsynaptic current (mIPSC) frequency and evoked IPSC amplitude in one-third of neurons, indicating that GLP-1 signaling facilitates presynaptic input in these neurons by acting on presynaptic GLP-1 receptors^17^.

GLP-1 receptors are abundantly expressed in the cerebellar cortex, including in the molecular layer, Purkinje cells (PCs), and the granular layer^26,27^. The cerebellar cortex receives many different types of afferent signals—including excitatory glutamatergic, inhibitory GABAergic, adrenocorticotropic, monoaminergic, and proglucagonergic projections—that are involved in the regulation of cerebellar neuronal activity^1,28^. Notably, afferents from nucleus tractus solitarius PPG neurons also project to all layers of the cerebellar cortex; however, the effect of GLP-1 on cerebellar cortical synaptic transmission has not yet been explored. We therefore studied the effects of GLP-1 on parallel fiber (PF)–PC synaptic transmission in mouse cerebellar slices.

## Material and methods

### Slice preparation

The experimental procedures were approved by the Animal Care and Use Committee of Yanbian University and were in accordance with the animal welfare guidelines of the U.S. National Institutes of Health. The permit number is SYXK (Ji) 2011-006. Cerebellar slices preparation has been described previously^29^. In brief, 4–6 weeks old ICR (Institute of Cancer Research) mice were deeply anaesthetized with halothane. After cutting the head of the mouse with scissors,, the whole brain was removed and placed in ice-cold artificial cerebrospinal fluid (ACSF) containing (in mM): 125NaCl, 3KCl, 1 MgSO_4_, 2 CaCl_2_, 1.25 Na_2_HPO_4_, 25 NaHCO_3_, and 10 D-glucose bubbled with 95% O_2_ and 5% CO_2_ (pH 7.4; 295–305 mOsm). The sagittal cerebellar slices (300 µm thick) were prepared using a Vibratome (VT 1200s, Leica, Nussloch, Germany). The slices were incubated over 1 h in a ACSF filled chamber at room temperature (24–25 °C) prior to patch-clamp recording.

### Electrophysiological recordings^29^

Whole-cell patch-clamp recordings from cerebellar PCs in slices were visualized using a 60 × water-immersion lens through a Nikon microscopy (Eclipse FN1, Nikon Corp., Tokyo, Japan). The composition of the recording electrode content is as follows (in mM): potassium gluconate 120, HEPES 10, EGTA 1, KCl 5, MgCl_2_ 3.5, NaCl 4, biocytin 8, Na_2_ATP 4, and Na_2_GTP 0.2 (pH 7.3 with KOH, osmolarity adjusted to 300 mOsm).Patch pipette resistances were 4–6 MΩ in the bath, with series resistances in the range of 10–20 MΩ. Membrane currents were monitored with an Axopatch 700B amplifier (Molecular Devices, Foster City, CA, USA), filtered at 5 kHz, and acquired through a Digidata 1440 series analog-to-digital interface on a personal computer using Clampex 10.4 software (Molecular devices, Foster City, CA, USA). Purkinje cells were held in voltage-clamp mode at -70 mV. Series resistance was monitored by injecting voltage pulses (10 ms, 5 mV), and only cells with stable series resistance were used for further analysis. Under the voltage-clamp recording mode, gabazine (20 µM) was added to the ACSF for blocking the GABAergic inhibition^29^. PF electrical stimulation was performed by a stimulating electrode containing ACSF (0.1–0.5 MΩ). The stimulating electrode was mounted on remote-controlled manipulators(MP-385, Sutter, Novato, CA, USA), and placed in the molecular layer of the cerebellar slice. Paired-current pulses (0.2 ms, 10–100 µA; duration: 50 ms; 0.05 Hz) were used for parallel fiber stimulation. For inhibiting postsynaptic PKA in some experiments, protein kinase inhibitor-(6–22) amide (PKI; 5 µM) was added in pipette internal solution^30^. Recordings of mEPSCs were performed in the presence of a mixture of gabazine (20 μM) and tetrodotoxin (TTX; 1 μM)^31^.

### Drug application

GLP-1, Exendin 9–39, TTX and gabazine were bought from Sigma-Aldrich (Shanghai, China). KT5720 and protein kinase inhibitor-(6–22) amide (PKI) were purchased from Tocris (Bristol, UK). KT5720 (1 mM) were diluted in dimethyl sulfoxide (DMSO). All the drugs were dissolved in solution and kept in frozen in aliquots, and they were applied to the cerebellar slices at 0.5 ml/min in ACSF through the peristaltic pump. When a stable whole-cell recording was formed, 5 min baseline was recorded before drugs application. In the experiments involving PKA inhibitor, KT5720 was applied at least 30 min before patch-clamp recording and continuing throughout the experiments.

### Statistical analysis

Electrophysiological data were analyzed using Clampfit 10.3 software. To evaluate the pre- or postsynaptic location of the GLP-1 modulated PF-PC synaptic transmission, the paired-pulse ratio (PPR) was calculated as the second EPSC amplitude over the first EPSC amplitude^32–35^.The frequency and amplitude of mEPSCs were analyzed using Mini Analysis software (Version 6.0.3; Synaptosoft, Decatur, GA). The original traces of mEPSCs were filtered digitally at 1 kHz. Only synaptic events showing a clearly defined baseline and a peak were used for amplitude analysis. During analysis, the threshold for detection of mEPSCs was set at 4pA and the period to search an mEPSC was set at 30 ms^36^.All the parameters were maintained constant for an individual recorded cell during treatments with ACSF, drugs and recovery. All data are expressed as the mean ± S.E.M. One-way and repeated measures ANOVA followed by Tukey's post-hoc test (SPSS software) were used to determine the level of statistical significance between groups of data. *P* values below 0.05 were considered to indicate a statistically significant difference between experimental groups.

### Ethics approval and accordance

The experimental procedures were approved by the Animal Care and Use Committee of the Yanbian University. The permit number is SYXK (Ji) 2011-006. All the experimental methods were in accordance with the animal welfare guidelines of the U.S. National Institutes of Health, and the Animal Research: Reporting in Vivo Experiments (ARRIVE; https://arriveguidelines.org).

## Results

### GLP-1 enhanced the parallel fiber stimulation-evoked EPSCs via GLP-1 receptor

**Figure 1:**
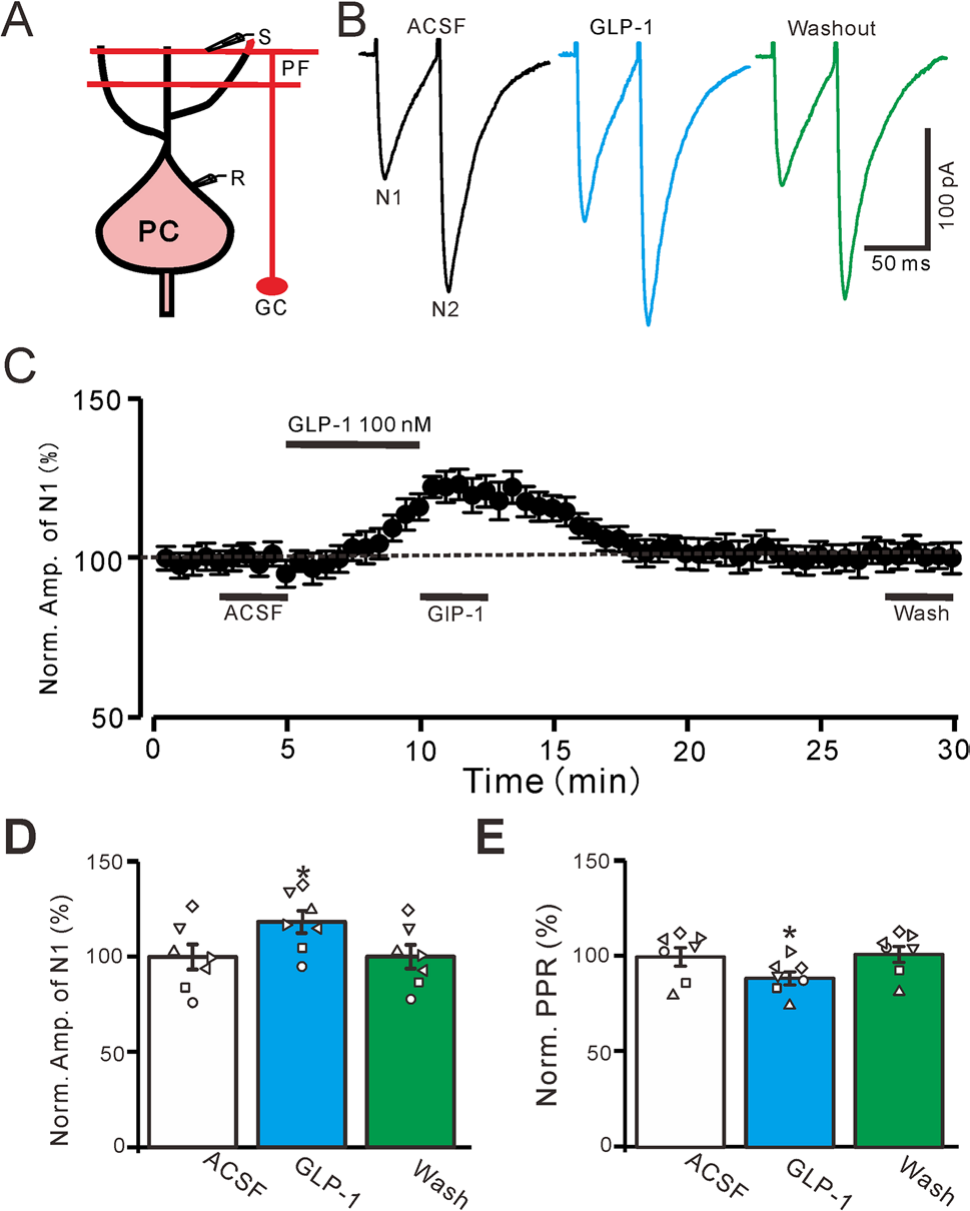
GLP-1 enhanced the PF stimulation-evoked EPSCs in mouse cerebellar slices. (**A**) A diagram showing the parallel fiber (PF) stimulation evoked EPSCs in a cerebellar PC. R, recording electrode; S, stimulation electrode; GC, granule cell. (**B**) Under voltage-clamp recording mode (Vh = -70 mV), the representative traces showing EPSCs (N1 and N2) evoked by a paired-pulse stimulation (0.2 ms, 10–100 µA; interval: 50 ms) during treatments of ACSF, GLP-1 (100 nM) and washout. (**C**) The mean (± S.E.M) value showing the time course of N1 during treatments of ACSF, GLP-1 (100 nM) and washout. (**D**) The mean (± S.E.M) and individual data showing the normalized amplitude of N1 during treatments with ACSF, GLP-1 and washout. (**E**) The mean (± S.E.M) and individual data showing the N2/N1 ratio during each treatment. *, P < 0.05 versus ACSF, n = 7 cells in each group.

Using the voltage-clamp recording mode (Vh =  − 70 mV), PF stimulation (0.2 ms, 10–100 µA; interval: 50 ms) evoked a pair of EPSCs that exhibited N1 and N2 (Fig. 1A,B). The bath application of GLP-1 (100 nM; 5 min) increased the amplitudes of N1 and N2 (Fig. 1B,C). In the presence of GLP-1 (100 s), the normalized amplitude of N1 was significantly higher than at baseline (118.6% ± 5.9% of the baseline; P = 0.026, n = 7, Fig. 1D). Because the PPR is a widely reported indicator of a presynaptic or postsynaptic locus of the mechanism^32–35^, we further analyzed the effects of GLP-1 on the PPR. Notably, GLP-1 application significantly decreased the PPR; the normalized PPR was 88.73% ± 3.41% of the baseline (P = 0.031, n = 7, Fig. 1E). These results indicate that GLP-1 increases PF-PC synaptic transmission through GLP-1 receptor activation.

**Figure 2:**
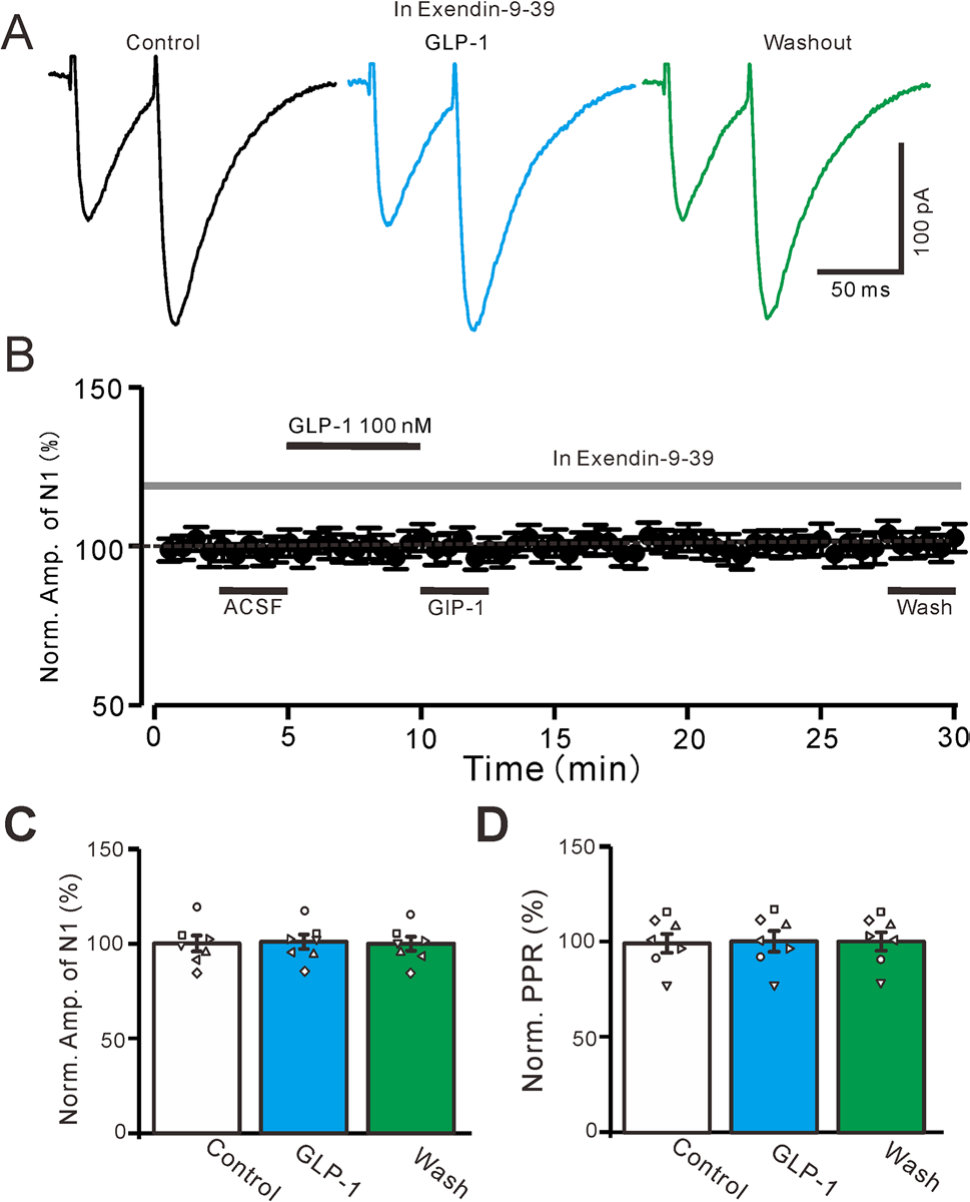
Blockade of GLP-1 receptor, GLP-1 failed to enhance the amplitude of PF-PC EPSCs. (**A**) In the presence of a GLP-1 receptor blocker (Exendin 9–39, 100 nM), the representative traces showing EPSCs (N1 and N2) evoked by a paired-pulse stimulation (0.2 ms, 10–100 µA; interval: 50 ms) under conditions of control, GLP-1 (100 nM) and washout. (**B**) In the presence of Exendin 9–39, the mean (± S.E.M) value showing the time course of N1 under control, GLP-1 (100 nM) and washout. (**C**, **D**) In the presence of Exendin 9–39, the mean (± S.E.M) and individual data showing the normalized amplitude of N1 (**C**) and the N2/N1 ratio (**D**) during each treatment. *, P < 0.05 versus control (Exendin 9–39), n = 7 cells in each group.

We then used a GLP-1 receptor blocker, exendin 9–39 (100 nM), to examine whether GLP-1 enhanced PF-PC EPSCs via GLP-1 receptors. Exendin 9–39 application completely prevented the GLP-1-induced enhancement of PF-PC EPSCs (Fig. 2A,B). In the presence of exendin 9–39 and GLP-1, the normalized amplitude of N1 was not significantly different from that of the baseline (exendin 9–39 alone) (101.2% ± 3.8% of the baseline; P = 0.67, n = 7, Fig. 2C). The normalized PPR was also similar to the baseline (101.11% ± 5.49% of the baseline; P = 0.64, n = 7, Fig. 2D). These results indicate that GLP-1 enhances PF-PC EPSC amplitude and decreases the PPR, suggesting that GLP-1 enhances PF-PF via GLP-1 receptor activation.

### PKA activation is required for the GLP-1induced enhancement of PF-PC EPSCs

**Figure 3:**
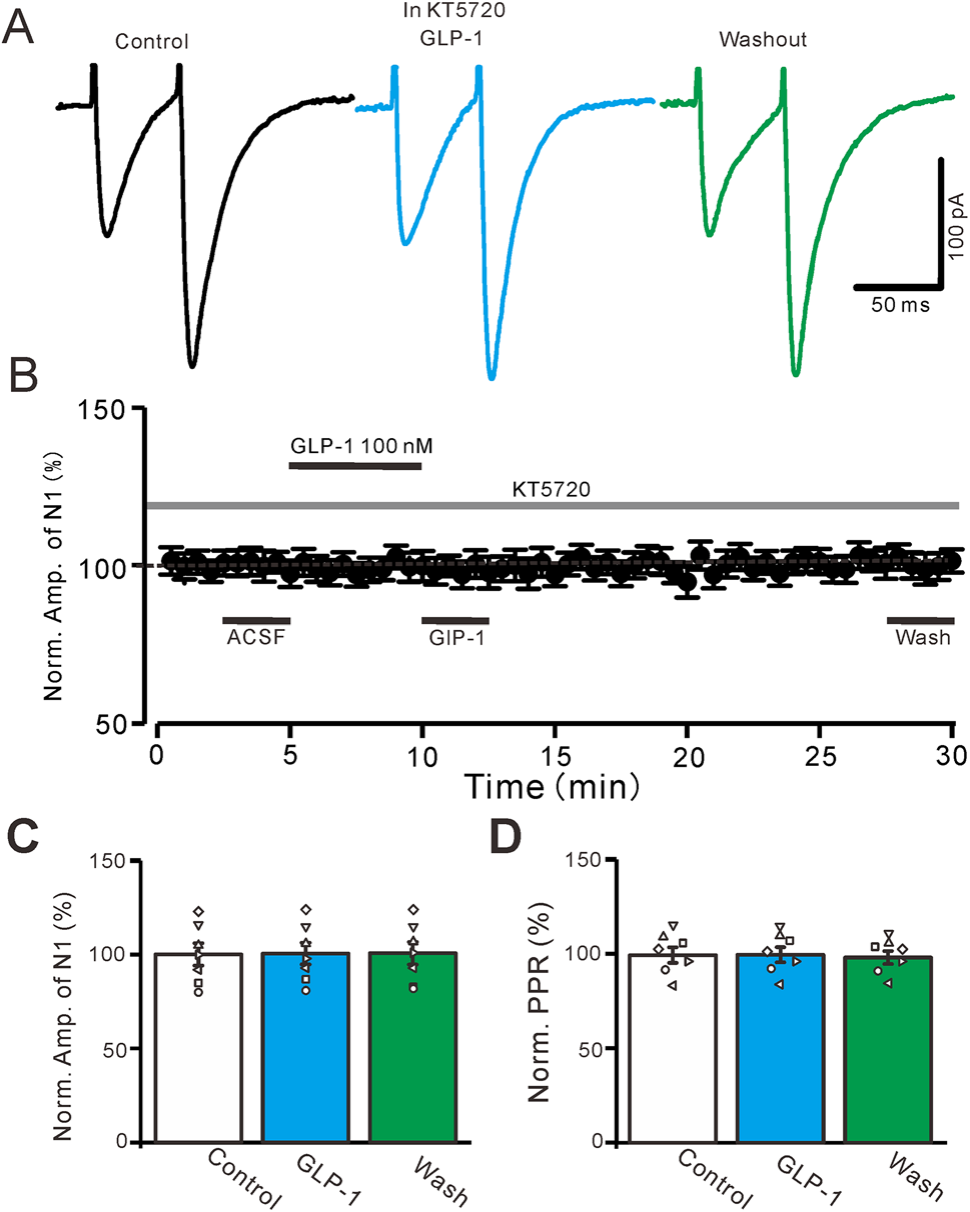
Inhibition of PKA abolished the GLP-1-induced enhancement of the eEPSCs. (**A**) The representative traces showing the EPSCs evoked by a paired-pulse stimulation (0.2 ms, 10–100 µA; interval: 50 ms) during treatments of KT5720 (500 nM), KT5720 + GLP-1 (100 nM), and washout of GLP-1. (**B**) In the presence of KT5720, the mean (± S.E.M) value showing the time course of N1 under control, GLP-1 (100 nM) and washout. (**C**, **D**) The mean (± S.E.M) and individual data show the normalized amplitude of N1 (**C**) and N2/N1 ratio (**D**) during treatments with KT5720, KT5720 + GLP-1, and washout of GLP-1. *, P < 0.05 versus control (KT5720), n = 7 cells in each group.

To evaluate the role of PKA in the GLP-1-induced enhancement of PF-PC EPSCs, we used a selective PKA inhibitor, KT5720 (100 nM). For the complete inhibition of PKA, KT5720 was added to the recording chamber with artificial cerebrospinal fluid (ACSF) for 30 min before whole-cell patch-clamp recording started. Consistent with previous studies^30,37^, PKA inhibition produced a decrease in PF-PC EPSCs as well as an increase in the PPR. In the presence of KT5720, the normalized amplitude of N1 was significantly lower than that at baseline (74.2% ± 3.9% of the baseline [ACSF only]; P = 0.003, n = 6) and the normalized PPR was significantly higher (111.2% ± 2.2% of the baseline; P = 0.026, n = 6; data not shown). In the absence of PKA activity, GLP-1 application failed to increase the PF stimulation-evoked EPSCs (Fig. 3A,B); in the presence of KT5720 and GLP-1, the normalized amplitude of N1 was 100.6% ± 5.8% of the control (KT5720 alone; P = 0.76, n = 7, Fig. 3C) and the normalized PPR was 100.15% ± 4.02% of the control (P = 0.68, n = 7, Fig. 3D). Together, these results indicate that PKA inhibition abolishes the effects of GLP-1 on PF-PC EPSCs, suggesting that the GLP-1 enhancement of PF-PF synaptic transmission is dependent on the PKA signaling pathway.

**Figure 4:**
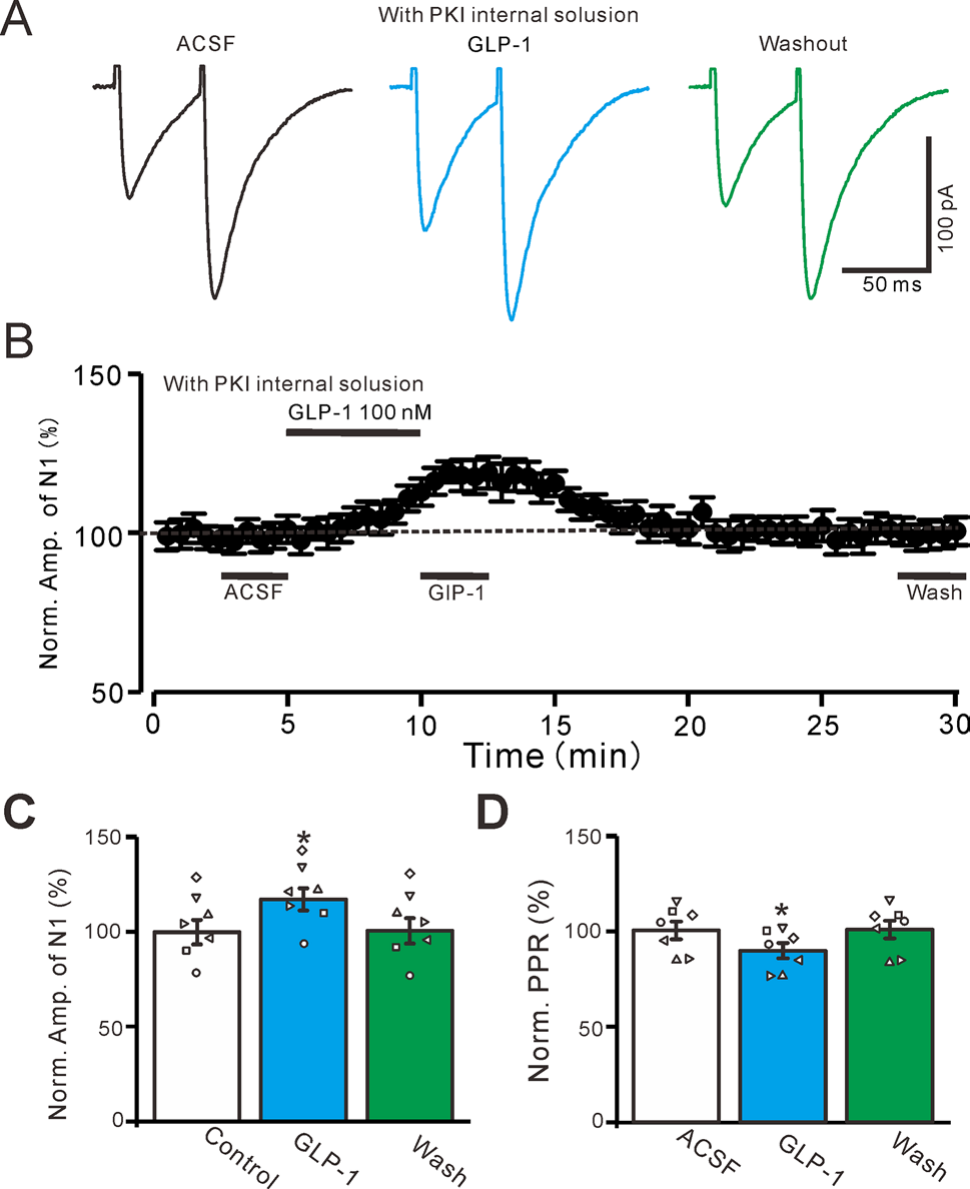
Blockade postsynaptic PKA, failed to prevent the GLP-1-induced enhancement of the PF-PC eEPSCs. (**A**) Using PKI containing pipette solution, the representative traces showing EPSCs (N1 and N2) evoked by a paired-pulse stimulation (0.2 ms, 10–100 µA; interval: 50 ms) during treatments of ACSF, GLP-1 (100 nM) and washout. (**B**) The mean (± S.E.M) value showing the time course of N1 during treatments of ACSF, GLP-1 (100 nM) and washout. (**C**) The mean (± S.E.M) and individual data showing the normalized amplitude of N1 during treatments with ACSF, GLP-1 and washout. (**D**) The mean (± S.E.M) and individual data showing the N2/N1 ratio during each treatment. *, P < 0.05 versus ACSF, n = 7 cells in each group.

To evaluate whether PKA antagonists act at the presynaptic terminal or the postsynaptic cell, a membrane-impermeable PKA inhibitor, PKI (5 μM), was added to the internal pipette solution^30^. With PKI in the internal solution, the bath application of GLP-1 (100 nM) increased the amplitudes of N1 and N2 (Fig. 4A,B). In the presence of GLP-1, the normalized amplitude of N1 was significantly higher than that of the baseline (117.3% ± 5.8% of the baseline; P = 0.031, n = 7, Fig. 4C) and the normalized PPR was significantly lower than that of the baseline (90.1% ± 4.01% of the baseline; P = 0.037, n = 7, Fig. 4D). These results indicate that the GLP-1-induced increase in PF-PC synaptic transmission is dependent on the presynaptic PKA signaling pathway, suggesting that GLP-1 upregulates glutamate release from PF terminals.

### GLP-1 enhances mEPSCs through GLP-1 receptors and PKA signaling

**Figure 5:**
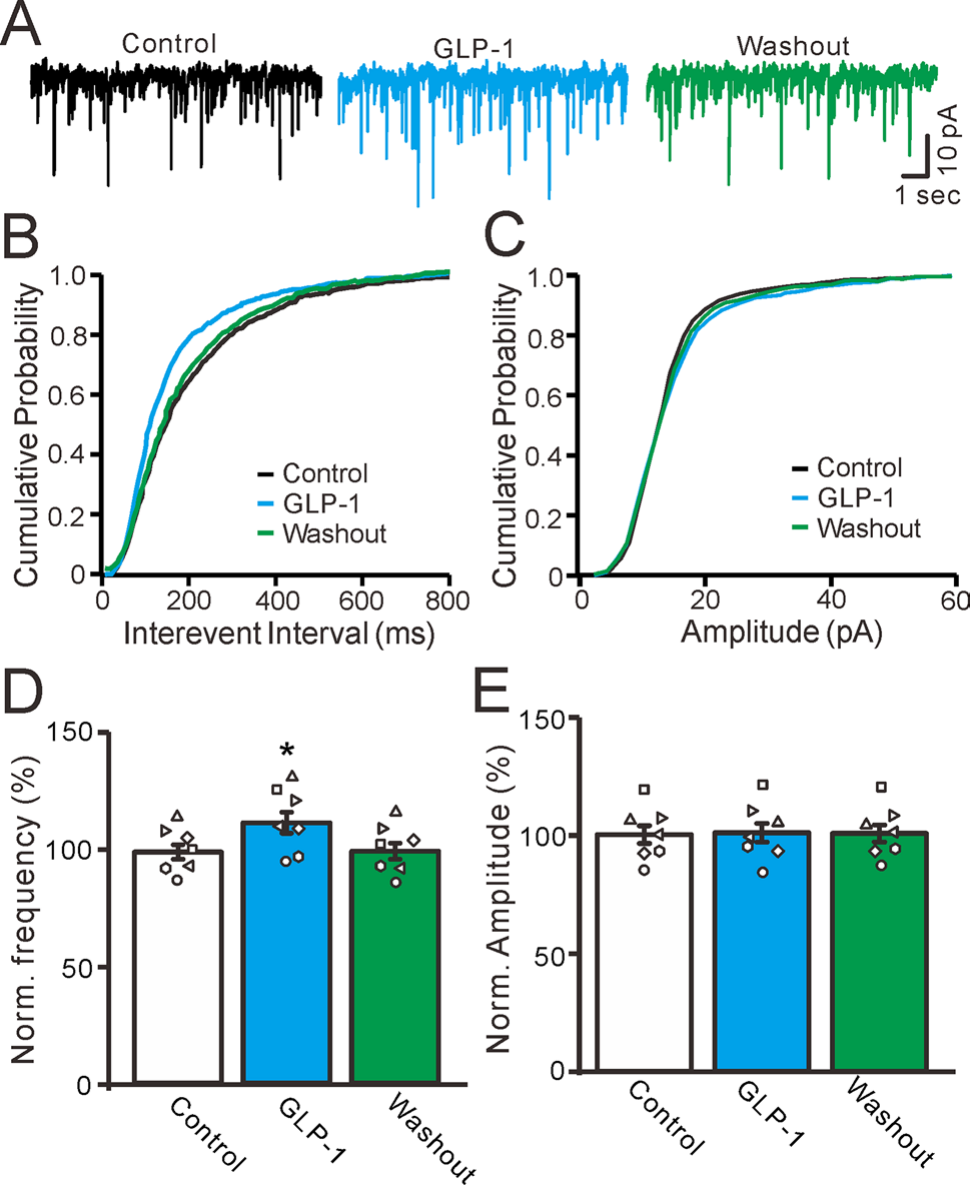
GLP-1 increased the frequency of mEPSCs in cerebellar PCs. (**A**) In the presence of gabazine(20 μM) and TTX (1 μM), representative membrane current traces of a cerebellar PC recorded in control, GLP-1 (100 nM) and washout. (**B**) Cumulative probability-interevent interval curve of mEPSCs in control, GLP-1 and washout. (**C**) Cumulative probability-amplitude curve of mEPSCs in control, GLP-1 and washout. (**D**, **E**) The mean (± S.E.M) and individual data show the normalized mEPSCs frequency (**D**) and amplitude (**C**) of the PCs in control, GLP-1 and washout. n = 7 cells in each group. * P < 0.05 versus control, n = 7 cells in each group.

The mEPSC is a widely used indicator of presynaptic sites and can be used to observe the presynaptic mechanisms of PF-PC synaptic transmission^32–35^. To determine whether GLP-1 affects glutamatergic synaptic transmission at PF terminals, we further examined the effects of GLP-1 on mEPSCs. To record mEPSCs from PCs, we added gabazine (10 μM) and TTX (1 μM) to ACSF to block spontaneous EPSCs and GABAergic inhibitory inputs. Under the voltage-clamp recording mode (Vh =  − 70 mV), GLP-1 administration increased mEPSC frequency (Fig. 5A), resulting in a leftward shift of the frequency-accumulation probability curve of mEPSCs (Fig. 5B); however, there was no shift in the amplitude-accumulation probability curve (Fig. 5C). In the presence of GLP-1, the normalized frequency of mEPSCs was 112.2% ± 4.6% of the baseline (P = 0.031, n = 7, Fig. 5D) and the normalized amplitude of mEPSCs was 100.9% ± 4.0% of the baseline (P = 0.73, n = 7, Fig. 5E). These results indicate that GLP-1 significantly increases mEPSC frequency but not amplitude, suggesting that GLP-1 increases glutamate release from PF terminals.

**Figure 6:**
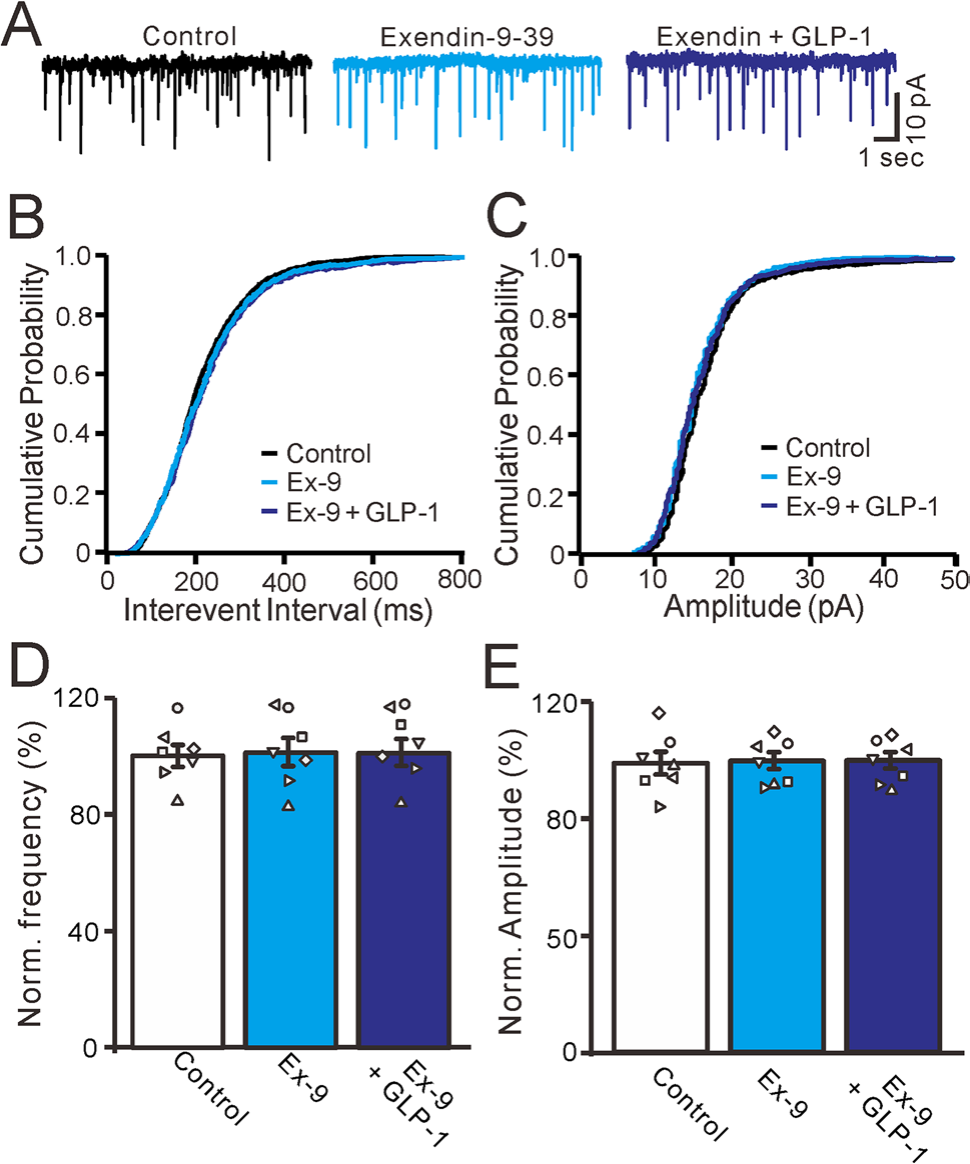
Blockade GLP-1 receptors, GLP-1 failed to change the frequency of mEPSCs in the cerebellar PCs. (**A**) In the presence of gabazine (20 μM)and TTX (1 μM), representative membrane current traces of a cerebellar PC recorded in control, Exendin 9–39 (100 nM) and Exendin 9–39 + GLP-1 (100 nM). (**B**) Cumulative probability-interevent interval curve of mEPSCs in control, Exendin 9–39 (100 nM) and Exendin 9–39 + GLP-1 (100 nM). (**C**) Cumulative probability-amplitude curve of mEPSCs in each treatment. (**D**, **E**) The mean (± S.E.M) and individual data show the normalized mEPSCs frequency (**D**) and amplitude (**C**) of the PCs in each treatment. n = 7 cells in each group.

We then used a GLP-1 antagonist to observe the effects of GLP-1 on mEPSCs in the absence of GLP-1 receptor activity. As shown in Fig. 6, mEPSC frequency and amplitude were not significantly affected by exendin 9–39 (100 nM) administration, but the effects of GLP-1 on mEPSCs were completely blocked (Fig. 6A). In the presence of exendin 9–39, GLP-1 administration did not lead to a significant increase in mEPSC frequency and there was no significant change in the frequency-cumulative probability curve of mEPSCs (Fig. 6B). Combined exendin 9–39 and GLP-1 administration also had no significant effects on mEPSC amplitude or the amplitude-cumulative probability curve (Fig. 6C). In the presence of exendin 9–39, mEPSC frequency was 101.2% ± 4.8% of the baseline (P = 0.63, n = 7, Fig. 6D). In the presence of both exendin 9–39 and GLP-1, mEPSC frequency was 100.8% ± 4.2% of the baseline, similar to the results with exendin 9–39 alone (P = 0.72, n = 7, Fig. 6D). In the presence of exendin 9–39, mEPSC amplitude was 100.6% ± 3.5% of the baseline (P = 0.76, n = 7, Fig. 6E). In the presence of both exendin 9–39 and GLP-1, mEPSC amplitude was 100.3% ± 3.5% of the baseline, similar to the findings with exendin 9–39 alone (P = 0.78 n = 7, Fig. 6E). Together, these results indicate that GLP-1 significantly increases glutamate release from PF terminals through its receptors, resulting in significantly increased mEPSC frequency.

**Figure 7:**
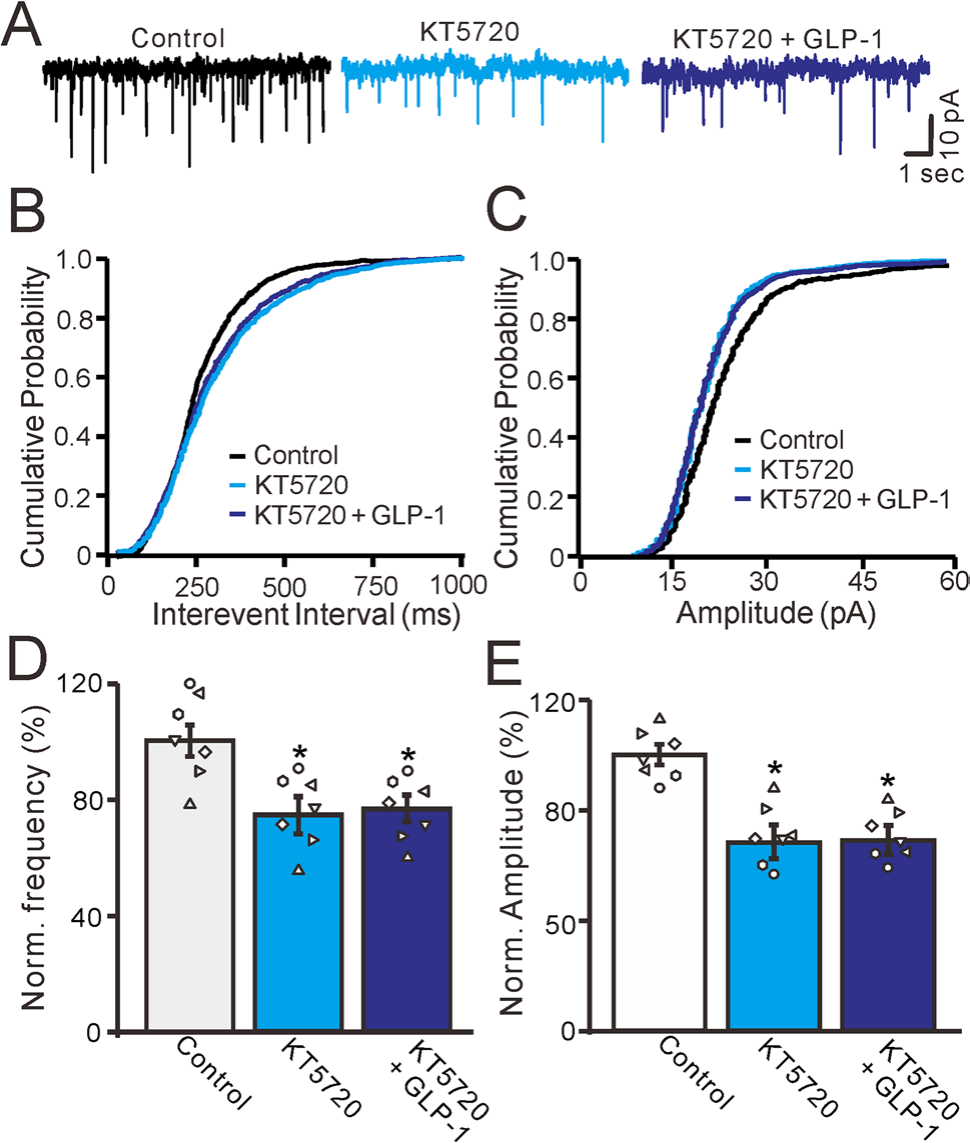
Inhibition of PKA, GLP-1 failed to change the frequency of mEPSCs in the cerebellar PCs. (**A**) In the presence of gabazine (20 μM) and TTX (1 μM), representative membrane current traces of a cerebellar PC recorded in control, KT5720 (500 nM) and KT5720 + GLP-1 (100 nM). (**B**) Cumulative probability-interevent interval curve of mEPSCs in control, KT5720 and KT5720 + GLP-1 (100 nM). (**C**) Cumulative probability-amplitude curve of mEPSCs in each treatment. (**D**, **E**) The mean (± S.E.M) and individual data show the normalized mEPSCs frequency (**D**) and amplitude (**C**) of the PCs in each treatment. * P < 0.05 versus control. n = 7 cells in each group.

Next, we used a specific PKA inhibitor, KT5720 (100 nM), to observe whether GLP-1 increased mEPSC frequency via the PKA signaling pathway. As shown in Fig. 7, after 30 min of KT5720 (100 nM) administration, mEPSC frequency was significantly reduced, and the frequency-cumulative probability curve of mEPSCs shifted to the right (Fig. 7A, B). In the presence of KT5720, GLP-1 did not increase mEPSC frequency and there was no significant change in the frequency-cumulative probability curve of mEPSCs (Fig. 7B). Furthermore, KT5720 application significantly decreased mEPSC amplitude and the amplitude-cumulative probability curve shifted to the left (Fig. 7C). In the presence of KT5720, GLP-1 application did not induce any further decreases in mEPSC amplitude, and the amplitude-cumulative probability curve did not change (Fig. 7C). After KT5720 was present for 30 min, the normalized frequency of mEPSCs was 74.6% ± 2.9% of the baseline (P = 0.006, n = 7, Fig. 7D). In the presence of KT5720 and GLP-1, the normalized frequency of mEPSCs was 74.7% ± 2.8% of the baseline, similar to the results with KT5720 alone (P = 0.76, n = 7, Fig. 7D). In the presence of KT5720, the normalized amplitude of mEPSCs was 65.6% ± 4.8% of the baseline (P = 0.002, n = 7, Fig. 7E). In the presence of KT5720 and GLP-1, the normalized amplitude of mEPSCs was 65.4% ± 4.6% of the baseline, similar to the findings with KT5720 alone (P = 0.74, n = 7, Fig. 7E). These results indicate that GLP-1 significantly increases glutamate release from PF terminals through the PKA signaling pathway, resulting in significantly increased mEPSC frequency in mice in vitro.

## Discussion

In the present study, GLP-1 increased the amplitude of PF stimulation-evoked EPSCs and decreased the PPR in cerebellar slices. The effects of GLP-1 on PF-PC EPSCs were abolished by both the bath application of a selective GLP-1 receptor antagonist and the extracellular administration of a PKA inhibitor. By contrast, the GLP-1-induced enhancement of PF-PC EPSCs was unaffected by the inhibition of postsynaptic PKA. Importantly, GLP-1 significantly increased mEPSC frequency; this effect was dependent on a GLP-1 receptor antagonist and PKA signaling. Together, these results indicate that GLP-1 receptor activation enhances glutamate release at PF-PC synapses via a presynaptic PKA signaling pathway, which results in enhanced PF-PC synaptic transmission in mice in vitro. These findings suggest that GLP-1 plays a critical role in the modulation of cerebellar function in living animals by regulating excitatory synaptic transmission at PF-PC synapses.

### GLP-1 regulates the PF-PC synaptic transmission through its receptors

Both PPG neuronal projections and GLP-1 receptors exist in all layers of the cerebellar cortex, suggesting that GLP-1 may have critical roles in regulating neuronal activity and synaptic transmission through the activation of its receptor^2,3,36^. Previous studies have demonstrated that GLP-1 receptor activation modulates neuronal activity and synaptic transmission in several brain areas^8,38–40^. The activation of GLP-1 receptors increases glutamate release leading to an increase in the spike firing rate of hippocampal CA1 neurons in rats in vitro^38^. GLP-1 receptor expression has been observed in whole-cell recordings from the bed nucleus of the stria terminalis, hippocampus, and PVN, where GLP-1 elicits a reversible inward current or depolarization^8^. In arcuate nucleus neurons, postsynaptic GLP-1 receptor activation leads to membrane potential depolarization, which is accompanied by an increase in spontaneous action potential frequency^39,40^. However, GLP-1 receptor activation produces inhibition or excitation in distinct subpopulations of neurons in the bed nucleus of the stria terminalis, suggesting that GLP-1 affects both excitatory and inhibitory synaptic transmission in mice in vitro^10^. Our present results indicate that GLP-1 increases PF-PC EPSC amplitude, suggesting that GLP-1 enhances PF-PC transmission via the activation of its receptor.

The PPR has been widely used in previous studies to investigate the presynaptic or postsynaptic location of mechanisms^32–34^. For example, in the lateral amygdala, kainite receptor activation inhibits the evoked EPSC amplitude but significantly increases the PPR, indicating that presynaptic kainite receptor activation inhibits glutamate release from neurons^32^. However, kainite receptor activation has also been reported to increase the evoked EPSC amplitude but decrease the PPR at PF-PC synapses in the cerebellar cortex, indicating that presynaptic kainite receptors upregulate glutamate release at PF-PC synapses^33,34^. In the current study, GLP-1 significantly decreased the PPR at PF-PC synapses, indicating that presynaptic GLP-1 modulates PF-PC synaptic transmission.

The activation of GLP-1 receptors significantly increases the frequency of spike firing and mEPSCs in PVN gonadotropin-releasing hormone neurons in mice in vitro^41^. Consistent with previous studies^12,41,42^, the present results indicate that GLP-1 significantly increases mEPSC frequency, suggesting that GLP-1 increases glutamate release at PF-PC synapses. Notably, the effects of GLP-1 on mEPSCs were abolished by the antagonism of GLP-1 receptors in our study, indicating that GLP-1 receptor activation significantly increases glutamate release from PF terminals, resulting in a significant increase in mEPSC frequency.

### GLP-1 regulates PF-PC synaptic transmission via the presynaptic PKA signaling pathway

GLP-1 regulates neuronal activity by modulating multiple signal transduction pathways, such as the ERK1/2, PKA, PKC, and cytosolic calcium signaling pathways^11,18^. The PKA signaling pathway plays a key role in regulating neurotransmitter synthesis and release, and GLP-1 receptor activation can affect the release function of glutamate and regulate the release rate of neurotransmitter vesicles through the cAMP–PKA signaling pathway^8,38^. Activated GLP-1 receptors couple to adenylate cyclase, resulting in increased intracellular cAMP and calcium concentrations; this further modulates neuronal signal transduction and gene transcription and induces AMPK inhibition and MAPK activation of neurons^21^. In the central nervous system, glutamate and GABA release are regulated by various presynaptic modulators. At cerebellar PF-PC synapses, presynaptic kainate receptor activation modulates neurotransmitter release through an adenylate cyclase–cAMP–PKA signaling pathway^33–35^. In the current study, PKA inhibition abolished the effects of GLP-1 on PF-PC EPSCs, suggesting that GLP-1 enhances PF-PF through activation of the presynaptic PKA signaling pathway. Our results are consistent with those of previous studies^12,42^; for example, GLP-1 receptor activation in PVN corticotropin-releasing hormone neurons enhances AMPA receptor function and excitatory glutamate synaptic transmission via the PKA signaling pathway^42^. Moreover, GLP-1 activation induces resting membrane potential depolarization and increased spike firing frequency in ganglion cells via the cAMP pathway^12^. Importantly, we added a membrane-impermeable PKA inhibitor to the internal pipette solution in the current study, to examine whether PKA antagonists act at the presynaptic terminal or the postsynaptic cell. Blockade of postsynaptic PKA failed to prevent the GLP-1-induced enhancement of PF-PC EPSCs, thus confirming that GLP-1 increases PF-PC synaptic transmission via the presynaptic PKA signaling pathway. Moreover, GLP-1 failed to increase mEPSC frequency, indicating that GLP-1 increases glutamate release from PF terminals through the presynaptic PKA signaling pathway in mice in vitro.

GLP-1 receptors are G-protein coupled receptors that play important roles in regulating neuronal activity and synaptic transmission via cellular signaling pathways^8,10,43^. Consistent with previous studies^43,44^, our results suggest that GLP-1 binds to its receptor and enhances adenylate cyclase activation at PF terminals; this leads to increased intracellular cAMP levels and PKA activity, thus resulting in increased PF-PC synaptic transmission. The present study provides novel insights into the regulatory function of GLP-1 in the central nervous system. Furthermore, they suggest that GLP-1 influences cerebellar PC output in living animals, thus affecting motor coordination and motor learning function.

## Data Availability

The datasets generated and analyzed during the current study are available from the corresponding author on reasonable request.

## Abbreviations

GLP-1: Facilitates
PF: Glutamate release
